# Supportive Outpatient‐Provider Communication—A Scoping Review About Expectations, Experiences, and Actual Behaviours Regarding Healthcare Visits

**DOI:** 10.1111/jep.70626

**Published:** 2026-10-11

**Authors:** Sari Knaapi‐Junnila, Tuula‐Riitta Välikoski, Pekka Isotalus

**Affiliations:** ^1^ Tampere University Tampere Finland; ^2^ University of Turku Turku Finland

**Keywords:** communication, expectations, experiences, healthcare, outpatients, social support, supportive communication

## Abstract

**Rationale:**

Twenty‐first century healthcare has struggled with chronic illnesses while policies have emphasised patient centeredness and support. The importance of ambulatory visits and self‐management necessitate understanding about supportive outpatient‐provider communication.

**Objectives and Methods:**

This systematic scoping review focused on supportive communication as actual behaviours, and outpatients' expectations and experiences concerning visits. CINAHL, CIOS ComAbstracts, CMMC, and MEDLINE were searched for answering questions about (1) the extent, range, and nature of studies and (2) existing knowledge in 21st century. The framework presented by Arksey and O'Malley and the PRISMA Extension for Scoping Reviews were utilised.

**Results:**

After screening 310 hits, twelve studies were included. Studies were usually quantitative surveys about experiences, cancer, and specialised physicians in US. Existing data suggest that patients share common ground regarding supportive communication preferences while also differences exist. Access to care and it's continuity include in support desires. Provider's and patient's qualities affect provided support. Education and health status, inter alia, affect experiences. Mismatch between expected and experienced support, and experience of non‐constructive support include in findings.

**Discussion and Conclusion:**

This review contributed by summarising the existing studies and findings, and by identifying research gaps. Actual evidence about supporting outpatient‐provider communication is still thin and unilateral. Thus, gaps are manifold and we must be cautious with interpretations. Interdisciplinary collaboration is required to enhance our understanding. The lack of studies concerns especially (a) qualitative studies about diverse cultures, patients, and providers in primary care; (b) video materials about actual behaviours; (c) expectations (as values) together with experiences; (d) studies about expectations as probabilities. Importantly, organisations/systems should be perceived as integral (f)actors of supportive outpatient‐provider communication. Existing knowledge about supportive, person‐centred, and patient‐provider communication is valuable until we know more about supportive outpatient‐provider communication.

## 1 Introduction

The 21st century has entailed a dramatic, global increase in ageing populations with chronic conditions [1, 2, 3, 4, 5]. Globally, one in three of adults suffer from more than one chronic condition [3], and in the European Union, approximately 95% of the primary care population aged 65 years or older have multimorbidity [4]. When living with chronic illnesses, patients' needs differ significantly from those having acute diseases [6, 7, 8, 9, 10, 11, 12]. However, raising healthcare costs [2, 3, 13] and major reforms [1, 2, 12, 14, 15, 16, 17] have transformed healthcare entirely. In EU, the number of hospital beds and the average length of stay as inpatients has decreased considerably since 2000 [18] while care has fragmented [12] in rushed [19] outpatient settings. Thus, the role of ambulatory visits has expanded and emphasised alongside increased pressures see [12].

Simultaneously, patient/person/people‐centeredness with its core elements of communication and holistic support [1, 20] has become established as one of the dominant paradigms in Western countries [11, 21] and globally recommended practice [1, 2, 22]. The necessity of support—especially for self‐management—is emphasised both in research [23, 24, 25, 26] and in healthcare policies globally [27, 28, 29, 30]. Broadly, the purpose of support is to '“*manage well with condition(s)”* [31, 32]. However, healthcare still struggles with its' implementation [12, 20, 33, 34, 35, 36, 37]. Further, customising supportive communication behaviours for long‐term patients in rushed clinics provokes practical and ethical tensions, and uncertainties [38]. Shortness of time [19], superficial interaction processes, and scattered encounters [12, 36, 37] suggest that supportive communication is not necessarily available despite of potential expectations, healthcare guidelines, and well‐known benefits. Therefore, it is reasonable to say that not only chronic illnesses and healthcare reforms but also shortcomings in implementing patient‐centredness emphasise the relevance to study supportive outpatient‐provider communication.

Communicating social support is an integral part of human behaviour [39, 40]. In healthcare, it is a central part of providers' work with patients [41, 42]. Hence, we can suppose that supportive communication should actualise—at least somehow and to some extent—in outpatient visits. As a concept, *social support* refers to connections between social relationships and well‐being [43], whereas s*upportive communication* refers to the manifestation of verbal or nonverbal social support interpersonally [44]. Here, we focus on *supportive outpatient‐provider communication in physical visits*.

Communication is action with purpose. When providers communicate with patients, they may have multiple goals and motivations. Providing support may include in them, as supportive communication is known to affect both psychological and physical health [45]. Support is provided as an intention to assistance somebody, who is perceived as needing that aid [45] to improve well‐being in problematic, distressed, situation [46]. It can be provided in several ways and more or less skilfully [45, 47, 48]. The high incidence of ineffective or insensitive ‘support’ suggests that failing is common [45].

Providers can support for instance by assisting patients in disease‐related problems (problem‐solving support) [49, 50]; expressing acceptance, listening openly, encouraging to dialogue and questions (esteem support); expressing caring and empathy (emotional support); providing relevant information and advice for self‐management by comprehensible concepts (information support); offering medical referrals and medicine prescription (tangible support); and giving a hint about support groups (network support) (see e.g., [43, 45]).

It is well known that individuals may gain numerous benefits for their health, well‐being, and coping by communication [51] and receiving support [39, 45, 52]. The mechanisms are many while some of the connections between communication and support to positive health effects are more direct (e.g., providing health‐related information and reducing emotional distress) than others (e.g., promoting healthy behaviour and self‐management) [45, 51].

We also know considerably about the methods to communicate support. For example, problem‐solving support (such as advice, aid, information, offer, request) is useful for controllable problems [46]. Further, advice is more effective if it begins with emotional support and engagement in problem analysis, until given (if desired) politely with arguments and face‐work [45, 53]. Emotional support is more effective when feelings are explicitly acknowledged, elaborated, and legitimised by highly person‐centred messages. However, outcomes are affected by several factors (such as provider, receiver, message, and context) [47, 54, 55]. Consequently, well‐meant support is not necessarily *experienced* as (qualitatively or quantitatively) adequate, desired, or helpful [45, 56], but rather ineffective, unwanted, or even disadvantageous [45, 57].

Patients' expectations have been studied for multiple decades by several disciplines with diverge interests, definitions, conceptualisations, and methods [58, 59, 60]. In 20th century they were studied especially as probabilities and values relating to either a visit or ongoing care [59]. Knowledge about patients' perceptions that given behaviours are wanted/desired See [59, 61] expectations as values—is essential as communication should be appropriate and suit the receiver's needs, culture, and situation to be evaluated as supportive [45, 57]. Likewise, knowledge about patients' future‐directed beliefs—expectations as probabilities—is necessary when aiming better outcomes because of their predictive nature [62]. The influence of patients' expectations on physical and mental health, and medical treatment outcomes is notable [62, 63]. When assessing experiences, patients evaluate how their (initial) expectations are responded [64, 65]. Patients' experience, in turn, is acknowledged as an independent dimension of healthcare quality [66] and essential part of patient‐centred healthcare [1].

There are considerably studies about patient‐physician communication, social support, and supportive communication. However, knowledge about *supportive patient‐provider communication in outpatient visits*—especially the *communication perspective* [45] is quite rare. Considering commonness of ambulatory care and chronic illnesses, and the obtainable benefits that supportive communication could bring for individuals and societies, knowledge about actual outpatient clinic visits as for expectations and experiences concerning it, is required.

The aim of this review [67, 68] is to investigate *supportive patient‐provider communication* in outpatient visits both as behaviours and patients' expectations and experiences. We contribute to existing knowledge by summarising the studies and findings for decision makers and practitioners, and by identifying research gaps for researchers. We examine *supportive patient‐provider communication as actual behaviours in* (physical) *outpatient clinic visits, and as patients' expectations and experiences from the communication perspective of social support* [45]. Regarding behaviours, we focus on *supportive, interpersonal communication as such*. As for expectations and experiences interest us in every sense (e.g., as values and probabilities). In all, communication is seen as a situated process affected by a variety of social contexts [69]. The research questions to be answered are as follows:

What is the extent, range, and nature of research about *supportive* patient‐provider *communication* studied in outpatient clinic visits either as actual behaviours or patients' expectations and/or experiences?

What is known about *supportive* patient‐provider *communication* in outpatient clinic visits either as actual behaviours or patients' expectations and/or experiences?

## 2 Methods

### 2.1 A Systematic Scoping Review

We selected the review type based on the research aim and its' relativist assumptions. A scoping review is applicable for answering complex questions with heterogenic sources of evidence [67, 70, 71, 72, 73, 74, 75]. It is useful when aiming to arrange and interpret information, seek variation, and understand research within its context instead of statistical aggregation [75]. It is both systematic and transparent method aimed to reinterpret studies analytically and provide a narrative overview without appraisal [67, 70, 76]. The framework presented by Arksey and O'Malley [67] provided us an appropriate method for mapping, determining, and identifying the extent, nature, and range of relevant literature with useful findings ([70, 76]). Along with this iterative and reflexive five‐stage process (Subsections 2.2–2.4) [67] we utilised the PRISMA Extension for Scoping Reviews [68].

### 2.2 Stages 1–2: Identifying the Research Questions and Relevant Studies

We started the review from identifying two research questions (Section 5) as our interest was both in characteristics of studies, and their findings. We settled the focus on study articles published in English in peer‐reviewed academic journals. Then, we set a time frame from the beginning of the year 2000 to the end of October 2021. This time frame is based on the major changes in society and healthcare, and the person‐centeredness paradigm which acknowledged the value of support as an integral part of patient‐provider communication [77].

Research about support and patient‐provider interaction is conducted across multiple disciplines and perspectives. We aimed at studies done about physical outpatient clinic visits specifically *from the communication perspective of social support*—supportive patient‐provider communication—not in sociological or psychological perspectives [45]. Thus, after consultation of university informatician and preliminary searches we aimed to cover primarily communication, health, medical, and nursing sciences by four databases: CINAHL, CIOS ComAbstracts, Communication & Mass Media Complete (CMMC), and MEDLINE. We tailored each search uniquely because of database differences. Likewise, while using EBSCO, each search run in CINAHL, CMMC, and MEDLINE was separate. Along the process we reassessed the implications of chosen concepts repeatedly and redefined the research questions. Finally, after throughout preliminary searches targeting Boolean search to supportive communication with only mild limiters and expanders proved to provide the most relevant studies. The employed search strategies were broad, as recommended [67] (Table 1).

**Table 1:** Database searches.

Database	Search
CINAHL (EBSCO)	*Boolean/Phrase*: TX supportive communication OR AB support AND AB communication
	*Expander*: Apply related words
	*Limiters*: Published Date: 20000101‐20211031, English Language, Peer Reviewed, Human, Language: English, Publication Type: Journal Article, Outpatients, Age Groups: All Adult
CIOS ComAbstracts	*Phrase*: supportive communication (title & abstract)
	*Expander*: Allow synonyms
	*Limiters*: All sources, Years 2000–2021
Communication & Mass Media Complete (CMMC)	*Boolean/Phrase*: AB ‘supportive communication’
	*Limiters*: Scholarly (Peer Reviewed) Journals, Published Date: 20000101–20211031, Publication Type: Academic Journal, Document Type: Article, Language: English
MEDLINE (EBSCO)	*Boolean/Phrase*: AB (‘supportive communication’)
	*Expander*: Apply related words
	*Limiters*: Scholarly (Peer Reviewed) Journals, Date of Publication: 20000101–20211031, English Language, Human, Age Related: All Adult: 19+ years, Publication Type: Journal Article, Language: English

### 2.3 Stage 3: Study Selection

In this stage, we confirmed certain aims, settings, patients, and providers for inclusion and exclusion criteria. We included both primary and secondary care. As outpatients with notable support needs typically visit physicians, nurses, and physiotherapists [78], we chose them for providers with adults as target patients. We did not require that studies would focus mainly on supportive communication. Further, we did not suppose that the studies concern *only* outpatient setting(s) or providers if information for the research questions existed. See study flow diagram about the selection process with detailed inclusion and exclusion criteria (Figure 1).

**Figure 1:**
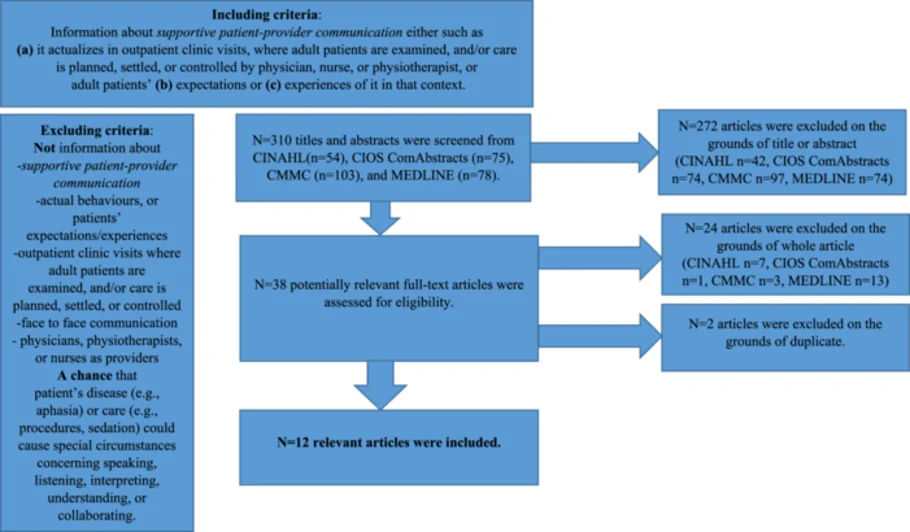
Study flow diagram about the selection process.

### 2.4 Stages 4–5: From Data Charting to Collating, Summarising, and Reporting

When charting the data, we managed information with a multipage table system on Excel. First, we apprised each database hit by title and abstract. When one or more exclusion criteria were met, we judged studies to ‘no’ (excluded) or ‘maybe’ category (for further exploration). Second, we reviewed ‘maybe’ category by reading full texts and judging either ‘no’ (excluded) or ‘yes’ (included). Each exclusion resulted usually from several unfulfilled criteria. We documented these reason(s) and re‐evaluated carefully during the process. Third, we merged ‘yes’ categories from all databases and duplicates were excluded. Supporting data about studies were extracted, collated, and saved on Excel. We have utilised the information from all these stages when summarising and reporting the results, which follow next.

## 3 Results

In this section, we will answer the research questions separately. The first question will be answered in Subsection 3.1. and the second in Subsection 3.2.

### 3.1 The Extent, Range, and Nature of the Research in Question

In this subchapter, we present publications and studies by extent, range, and nature. The final search strategy generated 310 hits from four databases. First, we read titles and abstracts. After this stage, we excluded 272 studies. Then, we read fully the remaining 38 articles and assessed them for eligibility. From those, we excluded 24 based on excluding criteria, and two as duplicates. Hence, 12 studies met the inclusion criteria making them relevant. See the study flow diagram with criteria (Figure 1). The included studies covered 21st century. MEDLINE and CINAHL served best for this topic as databases, while healthcare related journals proved to be main publishers for these studies accompanied only one communication journal. See the included publications [56, 79, 80, 81, 82, 83, 84, 85, 86, 87, 88, 89] (Table 2).

**Table 2:** Included publications by year, publication details, and databases.

Year	Publication	Journal and other publication details	Database(s)
2002	Chelf JH; Deshler AMB; Thiemann KMB; Dose AM; Quella SK; Hillman S. Learning and support preferences of adult patients with cancer at a comprehensive cancer centre.	Oncology Nursing Forum, Jun 2002; 29 (5): 863–867.	CINAHL
2002	Curtis JR; Wenrich MD; Carline JD; Shannon SE; Ambrozy DM; Ramsey. Patients' perspectives on physician skill in end‐of‐life care: differences between patients with COPD, cancer, and AIDS.	CHEST, Jul2002; 122 (1): 356–362.	CINAHL
2002	Westaway MS; Rheeder P; van Zyl DG; Seager JR. Development and testing of a 25‐item patient satisfaction scale for Black South African diabetic outpatients.	Curationis, Aug2002; 25 (3): 68–75.	CINAHL
2005	Street Jr RL; Gordon HS; Ward MM; Krupat E; Kravitz RL. Patient participation in medical consultations: why some patients are more involved than others.	Medical care, Oct2005; 43 (10), 960–969.	MEDLINE
2006	Street Jr, RL.; Gordon, HS. The clinical context and patient participation in post‐diagnostic consultations.	Patient Education & Counselling, Dec2006, 64 (1–3), 217–224.	CMMC & MEDLINE
2012	Morken, IM.; Norekvål, TM.; Bru, E; Larsen, AI.; Karlsen, B. Perceptions of healthcare professionals' support, shock anxiety and device acceptance among implantable cardioverter defibrillator recipients.	Journal of Advanced Nursing, Jan2012; 70 (9): 2061–2071.	CINAHL
2014	High, AC.; Steuber, KR. An Examination of Support (In)Adequacy: Types, Sources, and Consequences of Social Support among Infertile Women.	Communication Monographs. Jun2014, 81 (2),157–178.	CMMC
2015	Shumway D; Griffith KA; Jagsi R; Gabram SG; Williams GC; Resnicow K. Psychometric properties of a brief measure of autonomy support in breast cancer patients.	BMC medical informatics and decision making, 2015, 15:51.	MEDLINE
2016	Martinez KA; Resnicow K; Williams GC; Silva M; Abrahamse P; Shumway DA; Wallner LP; Katz SJ; Hawley ST. Does physician communication style impact patient report of decision quality for breast cancer treatment?	Patient Education and Counselling, Dec2016; 99 (12), 1947–1954.	MEDLINE
2017	López‐Sanromán, A; Carpio, D; Calvet, X; Romero, C; Cea‐Calvo, L; Juliá, B; Argüelles‐Arias, F. Perceived Emotional and Psychological Impact of Ulcerative Colitis on Outpatients in Spain: UC‐LIFE Survey.	Digestive Diseases & Sciences, Jan2017; 62: 207–216.	CINAHL
2018	Dean, M; Davidson, LG. Previvors' Uncertainty Management Strategies for Hereditary Breast and Ovarian Cancer.	Health Communication. 2018, 33 (2), 122–130.	CMMC & MEDLINE
2021	Zakaria M; Karim R; Rahman M; Cheng F; Xu J. Disparity in physician‐patient communication by ethnicity: evidence from Bangladesh.	International journal for equity in health, Feb2021; 20:65.	MEDLINE

Almost all the included studies were published in different journals while their publication time covered the settled timespan. Most studies were implemented in North America [56, 79, 80, 82, 83, 84, 87, 88], two in Europe [86, 89], one both in Africa [85], and Asia [81]. Further, in most studies patients were whites/Caucasians, and had cancer (seven out of 12). Usually, the data were from special clinics leaving primary care underrepresented with only two studies. Normally, only physicians (with varied experience and specialties) were studied. Physiotherapists were not mentioned, but nurses and some other providers were sometimes, although not without physicians. Visit durations ranged from less than 5 min up to 30 min (when reported). The data were collected from 2–4 (new) to 5–10 (existing) years before publication (when stated) (Table 3).

**Table 3:** Study characteristics—numerical summaries.

Characteristic	Studies, n; (*N* = 12)
Year of publication	
2002	3
2005, 2006, 2012, 2014, 2015, 2016, 2017, 2018, 2021	1 for each
Journal	
Patient Education & Counselling	2
BMC medical informatics and decision making, CHEST, Communication Monographs, Curationis, Digestive Diseases & Sciences, Health Communication, International journal for equity in health, Journal of Advanced Nursing, Medical care, Oncology Nursing Forum	1 for each
Continent, country (implementation & corresponding author's address)	
North America, US	8
Europe, Norway & Spain	2
Africa, South Africa	1
Asia, Bangladesh	1
Clinical setting(s) (different settings in some studies)	
Special clinics (at hospital or care centre)	11
Primary care	2
Diseases (diverse diseases in some studies)	
Cancer (during different stages—from genetic predisposition to end‐of‐life outpatient care)	7
Undefined	2
AIDS, bowel disease, cardiovascular disease, COPD, diabetes, infertility, and rheumatic diseases	1 of each
Patients	
Amount (range 34–1690, sometimes in multiple subgroups)	
Less than 100	2
100–499	7
500–999	2
1000–1999	1
Age	
Range 16–89 (based on cases, where reported exactly)	
Race/ethnicity	
All black	1
About half Bengali & half ethnic minority groups (in Bangladesh)	1
Mostly white (including Caucasian)	7
Not reported (Norway & Spain; probably mostly white)	2
Not reported (US)	1
Data	
Utilised the data from previous studies	4
Produced new data for the study in question	8
Type of data (diverse in some studies)	
Audio‐recordings (appointments)	2
Audio‐recordings (focus groups)	1
Audio‐recordings (phone interviews)	1
Individual interviews (personal, phone)	2
Questionnaires/surveys (online, phone, post‐consultation, posted)	8
Type of supportive communication	
Patients' expectations (alone)	3
Patients' experiences (alone)	6
Patients' expectations and experiences (together)	1
Actual behaviours during interaction	2

Included studies were mainly quantitative. Half of them (six out of 12) concerned solely patients’ experiences, three expectations, one both, and two actual behaviours. Expectations and experiences were contextualised, conceptualised, and measured variously. However, expectations were always studied as behaviours valued by patients—not probabilities, norms, or either unformed or unmet issues [58, 59, 61]. *Experiences* were linked in society issues like culture, race, and socio‐economy status [81], and (health)care quality including patient‐centeredness [81, 88, 89], autonomy and self‐determination in behaviour change [87, 88], and empowerment [86]. Also, everyday impacts of chronic disease [89] and health status [85] and health‐related quality of life [89] were considered. Patients' perceptions about supportive communication concerned typically ongoing care—only once a single visit [81] (Tables 3, 4, 5).

**Table 4:** The (sociocultural) nature of studies with the utilised data sorted by perspective.

Study (year)	Sociocultural (micro) context: Clinical setting providers patients with diseases	Sociocultural (macro) context: Study location author affiliation/field journal	Data: Data year data type
**Studies about patients’ support expectations** (in all cases expectations were seen as values)			
Chelf et al. Learning and support preferences of adult patients with cancer at a comprehensive cancer centre (2002)	**Clinical setting**: A National Cancer Institute, outpatient care **Providers**: ‘Health‐care team’ (physicians & nurses) **Patients**: ‐ *n* = 625 adult outpatients with cancer ‐ Age (range) 22–87 years, median age 64, −54% female ‐ The time since diagnosis (range) 2 weeks to 32 years, median 1.9 years ‐ Education: 54% completed some postsecondary education. 10% had less than a high school education	**Study location:** North America, US **Author affiliation/field**: North America, US Cancer education, nursing, statistics **Journal**: Oncology Nursing Forum	**Data year:** Not known for sure **Data type**: Structured interviews, questionnaire
Curtis et al. Patients' perspectives on physician skill in end‐of‐life care: differences between patients with COPD, cancer, and AIDS (2002)	**Clinical setting**: End‐of‐life ‐outpatient care for patients with COPD, AIDS, or cancer Multiple medical institutions throughout the greater Seattle area, US **Providers**: Physicians with COPD/AIDS/cancer patients **Patients**: 79 outpatients: with COPD (*n* = 24), with AIDS (*n* = 36), with cancer (*n* = 19) **(1) Patients with COPD**: Oxygen‐dependent COPD Median age, y (range) 70,5 (54–78), female 16 (70%) Race/ethnicity, n (%) : white 23 (96%), African American 1 (4%) **(2) Patients with AIDS**: C3 AIDS as defined by the Centres for Disease Control and Prevention Median age, y (range) 41,5 (27–55), female 13 (36%) Race/ethnicity, n (%): white 17 (50%), African American 15 (44%), other than white, African American or Hispanic/mixed 2 (6%) **(3) Patients with cancer**: Metastatic cancer or non‐operable lung cancer Median age, y (range) 58 (30–70), female 12 (63%) Race/ethnicity, *n* (%): white 18 (95%), other than white, African American or Hispanic/mixed 1 (5%)	**Study location:** North America, US **Author affiliation/field**: North America, US Medicine & Nursing **Journal**: CHEST	**Data year**: From previous study year not known for sure (probably 2000) **Data type**: Audio‐recordings (focus groups)
Dean & Davidson. Previvors' Uncertainty Management Strategies for Hereditary Breast and Ovarian Cancer (2018)	**Providers:** ‘Healthcare providers’ **Patients**: 34 BRCA‐positive women [= individuals with a genetic predisposition to develop hereditary breast and ovarian cancer (HBOC), but who have not been diagnosed with cancer, are referred to as previvors.] Average age 38 years (range 27–67) Race/ethnicity: most (26,76%) Caucasian, one Chinese American, one Caucasian and Colombian, one Caucasian and Hispanic, five Caucasian and Ashkenazi Jew	**Study location:** North America, US **Author affiliation/field**: North America, US Communication **Journal**: Health Communication	**Data year**: 2014 **Data type**: Audio‐recordings (phone interviews)
**Studies about patients’ support experiences**			
Westaway et al. Development and testing of a 25‐item patient satisfaction scale for Black South African diabetic outpatients (2002)	**Clinical setting**: Diabetic outpatient visits at Pretoria Academic Hospital and Kalafong Hospital, South Africa **Providers**: Outpatient care ‘providers’ **Patients**: 263 black diabetic outpatients: 174 females and 89 males Age 16–89 years (average 53.5)	**Study location:** Africa, South Africa **Author affiliation/field**: Africa, South Africa Health & Medical **Journal**: Curationis	**Data year**: 1999–2000 **Data type**: Structured questionnaire
Morken et al. Perceptions of healthcare professionals' support, shock anxiety and device acceptance among implantable cardioverter defibrillator recipients (2012)	**Clinical setting**: Implantable cardioverter defibrillator (ICD) recipients attending follow‐up visits at an outpatient device clinic in a Norwegian university hospital. **Providers**: Physicians, nurses, and other healthcare professionals **Patients**: Implantable cardioverter defibrillator recipients (167) Age 22–85 years, mean age 64 years, 80% male 57 (34%) recipients had a history of shocks	**Study location:** Europe, Norway **Author affiliation/field:** Europe, Norway Health & Medicine & Education **Journal**: Journal of Advanced Nursing	**Data year**: 2010 **Data type**: (Posted) questionnaire
Shumway et al. Psychometric properties of a brief measure of autonomy support in breast cancer patients (2015)	**Clinical setting**: 2 cancer specialty centres at New York & Georgia, US **Providers**: Physicians: surgeons, medical oncologists, and radiation oncologists **Patients**: 235 women diagnosed with breast cancer within the previous 18 months Aged 20–79, mostly white	**Study location:** North America, US **Author affiliation/field**: North America, US Medicine & Public Health **Journal**: BMC Medical Informatics and Decision Making, open access	**Data year**: 2013 **Data type**: (Printed) questionnaire survey
Martinez et al. Does physician communication style impact patient report of decision quality for breast cancer treatment? (2016)	**Clinical setting**: Breast cancer care **Providers**: Physicians: breast cancer surgeons & medical oncologists **Patients**: 1690 women with breast cancer Race/ethnicity: 55% (*n* = 930) white, 17% (*n* = 287) black, 17% (*n* = 279) Latina, 9% (*n* = 156) Asian Mean age 61.5 years, range 25–83 years The majority (72%) had completed some college or more	**Study location:** North America, US **Author affiliation/field**: North America & Europe, US & Portugal Medicine & Public Health & Value‐Based Care Research & Human Kinetics **Journal**: Patient Education and Counselling	**Data year**: From a previous study, year 2013–2014 **Data type**: Survey in English or in Spanish
López‐Sanromán et al. Perceived Emotional and Psychological Impact of Ulcerative Colitis on Outpatients in Spain: UC‐LIFE Survey (2017)	**Clinical setting**: Ulcerative colitis patients’ follow‐up visits in hospital outpatient clinics in Spain **Providers**: Physicians: 39 gastroenterologists **Patients**: 436 patients with Ulcerative colitis (UC) Mean age 46 years, 53% male	**Study location:** Europe, Spain **Author affiliation/field**: Europe, Spain Medicine **Journal**: Digestive Diseases and Sciences (Dig Dis Sci)	**Data year**: 2014 **Data type**: Survey
Zakaria et al. Disparity in physician‐patient communication by ethnicity: evidence from Bangladesh (2021)	**Clinical setting**: Three district hospitals and three Upazila (sub‐district) hospitals in the Chittagong Hill Tracts area, Bangladesh (District hospitals are responsible for delivering secondary and primary‐care services. Upazila hospitals provide primary healthcare services.) Primary care medical consultations Normally a long line of patients waits for a physician to be seen in medical centres. Physicians are busy and deal with many patients. **Providers**: Bengali physicians, who work at district or Upazila hospitals **Patients**: The Bengali and ethnic minority patients (*N* = 850): 400 (47.1%) Bengali, 50 (52.9%) from three ethnic minority groups (in accordance with the overall population proportions of ethnicity in Bangladesh)	**Study location:** Asia, Bangladesh **Author affiliation/field**: Asia, Bangladesh & China Communication & Journalism, English, Public Health, Medicine **Journal**: International Journal for Equity in Health, open access	**Data year**: 2019 **Data type**: Structured post‐consultation questionnaire
**Studies about patients’ support expectations** (as values) **AND experiences** (together)			
High & Steuber. An Examination of Support (In)Adequacy: Types, Sources, and Consequences of Social Support among Infertile Women (2014)	**Providers:** ‘Medical professionals’ **Patients**: 301 infertile women Average age 35 years (range: 19–45 years) Relationship: approximately 9 years Infertile approximately 6.5 years 74% had actively tried to become pregnant within the past year Infertility due to a condition within the female (59.1%), the male (13.3%), both partners (9.3%), unexplained infertility (18.3%) Race/ethnicity: white (84.1%), black (8.6%), Asian (2.7), Hispanic (1%), multiracial (3.7%)	**Study location:** North America, US **Author affiliation/field:** North America, US Communication **Journal**: Communication Monographs	**Data year**: Not known for sure **Data type**: (Online) survey
**Studies about actual behaviours** (interpersonal supportive communication)			
Street Jr et al. Patient participation in medical consultations: why some patients are more involved than others (2005)	**Clinical setting:** **(1) Primary care** Visits scheduled for 10–20‐min **(2) Rheumatology clinics** Follow‐up visits, scheduled for 10–15‐min **(3) Oncology and thoracic surgery clinics** First visits to that physician, scheduled for up to 30 min **Providers**: (1) Physicians: 20 internal medicine and family medicine physicians, male & female (2) Physicians: Seven attending rheumatologists, four rheumatology fellows, male & female (3) Physicians: Four attending oncologists, two attending thoracic surgeons, 12 thoracic surgery fellows, male & female **Patients**: Mostly white, 20–86 years **(1) Primary care patients** ‐ *n* = 135, male & female ‐ had a new or worsening problem, or were at least ‘somewhat concerned’ about a serious undiagnosed condition **(2) Patients with systemic lupus erythematosus (SLE)** ‐ *n* = 79, all female **(3) Patients with a diagnosis of lung cancer or a pulmonary nodule** ‐ *n* = 65, mostly male	**Study location:** North America (US) **Author affiliation/field:** North America (US) Communication, Health services, Medicine **Journal**: Medical Care	**Data year**: **(1) Primary care patients**: From previous study, visit years not known for sure (probably 2000–2001) [90, 91, 92] **(2) Patients with systemic lupus erythematosus**: From previous study, visit years 1995–1996 [90, 93] **(3) Patients with lung cancer**: from previous study, visit years not known **Data type:** Audio‐recordings (appointments)
Street Jr & Gordon. The clinical context and patient participation in post‐diagnostic consultations (2006)	**Clinical setting:** **(1) Post‐angiography consultations (not included)**: Catheterisation laboratory within an hour after the catheterisation Relatively brief interactions, typically 5 min or less Physician and patient discuss the findings and decide on treatment. ‐ some patients (*n* = 19, 21%) received conscious sedation with midazolam, seven patients received an antihistamine, three patients received morphine **(2) Initial lung cancer visits**: Relatively long interactions, up to 30 min Physician and patient discuss the biopsy results and treatment options that may include supportive care, radiation, chemotherapy, and surgery. **Providers:** **(1) Post‐angiography consultations (not included)**: Physicians: Three cardiology fellows and four staff cardiologists (all male) **(2) Initial lung cancer visits**: Physicians: Four attending oncologists, two attending thoracic surgeons, nine thoracic surgery fellows, and two physicians assistants, males & females **Patients:** Mostly white **(1) Post‐angiography consultations (not included)**: Visited at a large Veterans Affairs Medical Centre, mostly male (*n* = 88/92) **(2) Initial lung cancer visits**: Visited at oncology and thoracic surgery clinics with diagnosis of lung cancer or a pulmonary nodule, mostly male (*n* = 62/65)	**Study location:** North America (US) **Author affiliation/field**: North America, US Communication, Health, Medicine **Journal**: Patient Education and Counselling	**Data year:** **Post‐angiography consultations data**: From a previous study, year 2001 [94, 95] **Lung cancer visits data**: From a previous study, years 2001–2004 [94, 96] **Data type**: Audio‐recordings (appointments)

**Table 5:** Included studies with their aims, methods, and findings related to scoping review objectives.

Study (year)	Aims, concepts and methods	Takeaways (at a more general and practical level) and findings (related to scoping review objectives)
Support expectations (as values in all cases)		
Chelf et al. Learning and support preferences of adult patients with cancer at a comprehensive cancer centre (2002)	Aims To identify content items for an inclusive education curriculum for adult patients with cancer, describe their learning and support preferences, determine the level of information provided to them, and assess the patients' interest in potential new services. Concepts (indicating expectations) Patients' preferences = > expectations as values Methods Multiple (mixed) methods: Phase I: Identification of core components of an inclusive patient education curriculum by a team. Phase II: Structured interviews. Phase III: Questionnaire (made, tested, and mailed). Statistical analyses.	Takeaway: Patients with cancer valued clear, accurate information about their diagnosis and interactive, interpersonal communication with physicians. Findings*: Cancer patients' preferences*: (1) discussions with physicians (66%), (2) discussions with nurses (34%), (3) brochures or booklets provided by physicians or nurses (33%), (4) self‐selecting print media from information displays (20%), and (5) talking with other patients with cancer (14%). Significant differences in preferences existed among subgroups (age, gender, and education).
Curtis et al. Patients' perspectives on physician skill in end‐of‐life care: differences between patients with COPD, cancer, and AIDS (2002)	Aims To examine the perspectives of patients with COPD, cancer, or AIDS regarding important aspects of physician skill in providing end‐of‐life care. Concepts (indicating expectations) Patients' perspectives regarding important aspects of physician skills = > expectations as values Methods Qualitative. Audio‐recorded, transcribed focus groups, content analysis based on grounded theory.	Takeaway: Physicians' ability to provide emotional support, communicate about end‐of‐life care, and secure both accessibility and continuity of adequate care, was important for end‐stage patients. Findings*: The importance of emotional support, communication, accessibility, and continuity was common for patients with advanced COPD, AIDS, and cancer*. Emotional support was the most frequently identified domain for each disease. Additionally, each disease group identified a unique, important theme. *Important for patients with COPD:* Information about end‐of‐life care. Information about diagnosis, disease process, treatment options and the mechanism of action of them, prognosis, what dying might be like, and advance care planning. Possibly the most essential information concerns the progressive and irreversible nature of severe COPD. *Important for patients with AIDS:* Pain control *Important for patients with cancer:* Maintaining hope despite a terminal diagnosis
Dean & Davidson. Previvors' Uncertainty Management Strategies for Hereditary Breast and Ovarian Cancer (2018)	Aim To examine how healthcare providers may assist previvors (individuals with a genetic predisposition to develop hereditary breast and ovarian cancer, who were not diagnosed) in uncertainty management regarding hereditary breast and ovarian cancer during health care encounters. Concepts (indicating expectations) How providers may assist in uncertainty management = > expectations as values Methods Qualitative. Audio‐recorded, transcribed phone interviews, coding, qualitative analyses.	Takeaway: Patients expected multiple types of support from healthcare providers to manage uncertainty. Providers can support, for example, by expressing their willingness to help, providing clear and detailed information, clarifying ‘known’ versus ‘unknown’ information, inviting & answering questions, checking understanding, summarising content, providing decision‐making partnership, offering to delay decision making, reviewing next steps, and providing additional resources (such as counselling services and support groups) to assist in managing uncertainties. Findings: *Previvors' uncertainty management strategies* regarding hereditary breast and ovarian cancer: (1) Seeking healthcare providers as informational sources, (2) Seeking healthcare providers as partners for decision making, (3) Seeking healthcare providers for supportive communication, and (4) Seeking referrals from healthcare providers for social support networks
Support experiences		
Westaway et al. Development and testing of a 25‐item patient satisfaction scale for Black South African diabetic outpatients (2002)	Aim To develop and test a 25‐item patient satisfaction scale for evaluating the quality of healthcare for black diabetic outpatients. Concepts (indicating experiences) Satisfaction, quality of healthcare Methods Quantitative. A structured questionnaire (developed and tested), statistical analyses.	Takeaway: Interpersonal supportive communication was valuable for outpatients—maybe even more in the case of mental distress. Supportive communication was experienced as health service quality. Findings: *Diabetic outpatients* found the interpersonal aspects (e.g., communication, consideration, encouragement, friendliness, helpfulness, and support) more important than the organisational aspects (logistics and technical expertise) for healthcare quality and their satisfaction. Diabetic patients with poor mental health were significantly less satisfied with the interpersonal quality of their care than those with good mental health.
Morken et al. Perceptions of healthcare professionals' support, shock anxiety and device acceptance among implantable cardioverter defibrillator recipients (2014)	Aim To examine the relationship between perceived support from healthcare professionals, shock anxiety, and device acceptance among implantable cardioverter defibrillator (ICD) recipients. Concepts (indicating experiences) Perceived (constructive and nonconstructive) support Methods Quantitative. A survey, posted questionnaires, statistical analyses.	Takeaway: Providing supportive communication, sufficient time, and information during visits were useful ways to help patients cope and get through the needed care despite of anxiety, as patients' anxiety can be prevented by supportive communication. Providers need to be aware that by non‐constructive communication (e.g., criticism, indifference, information that may lead to insecurity and fear) they may exacerbate stress and cause harm to patients. Findings: *Implantable cardioverter defibrillator recipients* gained advantages from perceived constructive support (such as providing understandable information, listening to patients' concerns, and allocating sufficient time to process emotions) from providers during regular follow‐up visits. This increased device acceptance, partly by constituting a buffer against shock anxiety. Patients' perceptions of nonconstructive support from providers may be a source of distress and can reduce device acceptance. The findings emphasise the importance of supportive communication for improving ICD acceptance.
Shumway et al. Psychometric properties of a brief measure of autonomy support in breast cancer patients (2015)	Aim To evaluate the psychometric properties of a modified brief version of the Health Care Climate Questionnaire (which measures patient perceptions of their clinician's autonomy supportive communication) adapted for breast cancer patients. Concepts (indicating experiences) Treatment experience, patient‐centred communication, perceptions of autonomy supportive communication Methods Quantitative. A print questionnaire survey (shortened, tested), statistical analyses.	Takeaway: Cancer patients differentiated their ratings of autonomy support according to each provider rather than lumped their experience into an overall rating. The Health Care Climate Questionnaire (mHCCQ) can be utilised to obtain patients' perceptions of how well physicians facilitate autonomous motivation and self‐regulated behaviour, thereby promoting more lasting behavioural change. The general perception of providers' autonomy supportive communication can be achieved by measuring only the overall score if the respondents' burden is a particular concern. However, a more accurate picture of patient experiences can be achieved by specialist‐specific ratings. Patients with more education may be more assertive and expect more from providers. Findings: *Breast cancer patients* perceived and reported differences in autonomy supportive communication across provider types when six questions of perceived physician autonomy support for surgeons, medical oncologists, and radiation oncologists separately, as well as overall treatment (by mHCCQ) were utilised.
Martinez et al. Does physician communication style impact patient report of decision quality for breast cancer treatment? (2016)	Aim To explore the association between patients' perceptions of autonomy supportive communication by surgeons and medical oncologists and the patient‐appraised quality of their breast cancer surgery and chemotherapy decisions. Concepts (indicating experiences) Communication style, perceived/preferred/received communication style, perceptions of autonomy supportive communication Methods Quantitative. A mailed survey, statistical analyses.	Takeaway: Autonomy‐supportive communication by cancer physicians improved patients' perceived decision quality. Overall satisfaction with the treatment decision process may be improved by utilising more patient‐centred care techniques, such as listening to and addressing patient needs and concerns, seeking their input, and supporting their autonomy in treatment decision making. Findings: Among *breast cancer patients*, autonomy‐supportive communication by physicians (measured by the Modified Health Care Climate Questionnaire, mHCCQ) was associated with higher subjective decision quality (decision regret, satisfaction, perceived adequacy of information to make the decision, sufficient time to make the decision, and level of decisional involvement, measured by the Brief Subjective Decision Quality Measure). Patient communication style preference ‐ either directive, (e.g., telling the patient what to do) or non‐directive (e.g., autonomy supportive) ‐ moderated the association.
López‐Sanromán et al. Perceived Emotional and Psychological Impact of Ulcerative Colitis on Outpatients in Spain: UC‐LIFE Survey (2017)	Aim To evaluate the perceived every day and emotional impact of ulcerative colitis (UC) on patients attending outpatient clinics and to explore patient–physician communication. Concepts (indicating experiences) Patient‐physician communication, physician behaviours perceived by patients, perceived emotional/psychological support Methods Quantitative. Questionnaire (UC‐LIFE, created), a survey handed by physicians, statistical analyses.	Takeaway: Chronic diseases (such as UC) may cause multiple negative emotional and social impacts regardless of disease severity. Patients may experience that emotional/psychological support is lacking, although healthcare providers would listen and ask about symptoms. Improved patient–provider communication, including also psychological, emotional, and social aspects could contribute better health‐related quality of life (HRQoL) in patients. Findings: *Patients with ulcerative colitis* had a high prevalence of emotional impacts even when they perceived a low symptomatic burden. They had feelings of depression and anxiety, a decrease in self‐confidence, lack of motivation, anger, depression, frustration, and embarrassment. Often, the emotional and psychological burden was not approached adequately during the appointments. Although most patients believed that their physician listened/asked about UC symptoms, many perceived that emotional/psychological support was lacking.
Zakaria et al. Disparity in physician‐patient communication by ethnicity: evidence from Bangladesh (2021)	Aim To explore whether physician‐patient communication differs by ethnicity in primary care medical consultations and to examine the factors influencing better communication behaviour in primary care medical consultations in Bangladesh. Concepts (indicating experiences) Satisfaction, favourable communication behaviour, patient‐centred behaviours, perceived supportive communication behaviours Methods Quantitative. A structured post‐consultation questionnaire (Physician‐patient communication behaviour scale, PPCB) right after the appointment (created, pre‐tested, translated), statistical analyses.	Takeaway: Using patient‐centred communication, increasing cultural competence, and reducing disparity in the socio‐economic status, and equity in the delivery of healthcare could be some solutions to improve and ensure that all patients receive high‐quality treatment and better health outcomes regardless of their race or ethnicity. Having a pre‐organised plan about the desired content of outpatient visits serve a useful way for patients to enhance communication. Findings: Concordant ethnicity between physician and patient was a key contributor to better patient‐centred communication behaviour. *Bengali patients* reported more supportive communication behaviours from Bengali doctors than ethnic minority patients. The difference of language and culture could be one explanation for that. Moreover, Bengali patients with higher level of education, and internet use experienced even better supportive provider–patient communication (possibly by having self‐efficacy to engage in communication with providers, thus influencing the dynamics) than the others. Talking time was one of the factors which associated significantly with better experiences in the case of *ethnic minority* patients. *In the case of all patients*, having a pre‐organised plan about the content of medical consultation was associated with the experience of better communication behaviour.
Support expectations (as values) & experiences (together)		
High & Steuber. An Examination of Support (In)Adequacy: Types, Sources, and Consequences of Social Support among Infertile Women (2014)	Aim To study gaps between the supportive communication people desire and receive in the context of infertility. Concepts (indicating expectations & experiences) Support (in)adequacy, amount, types, and gaps, desired and received supportive communication Methods Quantitative. An online survey, statistical analyses.	Takeaway: Healthcare providers need to understand that even they would provide emotional, network, tangible, and informational support, there may be gaps between desired and received support. Even providers' informational support may be inadequate. Findings: *Infertile women* experienced inadequacy both in the amount and types—emotional, network, tangible, and even informational—of support from providers. Moreover, these gaps were the most (compered to spouses, friends, family, and online sources).
Actual behaviours (interpersonal supportive communication)		
Street Jr et al. Patient participation in medical consultations: why some patients are more involved than others (2005)	Aim To examine the extent to which patient participation in medical interactions is influenced by (1) The patient's personal characteristics (age, gender, education, and ethnicity), (2) The physician's communication style (e.g., use of partnership‐building and supportive talk), and (3) The clinical setting (e.g., the health condition and medical specialty). Concepts (indicating actual support) Physicians' facilitative communication, verbal behaviours, communication/communicative style: partnership‐building (partnering behaviour) and other types of supportive communication (‐ talk) Methods Quantitative. Actual, audio‐recorded, transcribed, appointments, coding, statistical analyses.	Takeaway: Patient participation (asking questions, assertive responses, and expressions of concern or other negative emotions) depends on a complex interplay of personal, provider, and contextual factors. Clinical setting and provider's communicative style impact significantly on patient participation. Patient involvement could be facilitated by using more frequently partnership building and supportive communication. Findings: Physicians engaged in partnering behaviour at least once in 83% of the consultations (mean = 3.27 utterances; range = 0–25), but supportive talk in the form of praise, encouragement, reassurance, and empathy occurred in only 38% of the interactions (mean = 1.01 utterances; range = 0–19). Patients were more active participants the more their physicians used partnership building (i.e., verbal acts that either encouraged patient involvement or affirmed and accommodated the patient's active participation) and supportive talk (i.e., verbal behaviours that validated or supported the patient's emotional or motivational state). Physicians' supportive talk predicted patients' expressions of concern and assertiveness but was unrelated to question‐asking. Rather than being directly prompted by physician partnership‐building or supportive talk, patients' active participation behaviours were mostly patient‐initiated (84%). *Predictors of patients' active participation*: physicians' partnership‐building and supportive talk, clinical context, more education, ethnicity: white patients *Predictors of physicians' partnership building and supportive talk*: clinical context, female physicians, ethnicity: white patients
Street Jr & Gordon. The clinical context and patient participation in post‐diagnostic consultations (2006)	Aims To study how the clinical context affects patient involvement in medical encounters. Concepts (indicating actual support) Physicians' facilitative communication/talk, verbal behaviours, communicative style: partnership‐building (partnering) and supportive communication (‐ talk) Methods Quantitative. Actual, audio‐recorded, transcribed, appointments, coding, statistical analyses.	Takeaway: Clinical context (e.g., visit length, medical specialty, the health condition, and location of the visit) has a profound effect on patient participation. Regardless of the clinical setting, patient participation will likely be greater when proportionally more of the providers' communication is partnering and supportive. Therefore, patient involvement could be increased by using partnering and supportive communication. Findings: *Predictors of patients' participation:* clinical context, physician's facilitative talk, partnership‐building (e.g., asking for the patient's preferences, granting a patient's request, encouraging, or accommodating the patient's participation), supportive talk (e.g., statements of encouragement, reassurance, praise, and comfort), ethnicity: white patients *Predictors of physicians' facilitative talk:* clinical context, longer visits (amount, not proportion)

The studies, which focused solely on *patients' expectations* concerned AIDS, cancer, and COPD patients' preferences during end‐of‐life outpatient care [82], uncertainty management in the case of hereditary cancer [84], and cancer patients' learning and support preferences [83]. Their data were collected by focus groups [82], questionnaires [84], and phone interviews [83] (Tables 4, 5).

*Patients' experiences* were mostly studied by surveys. Couple of studies concerned measurement development. They dealt with patient satisfaction scale (with interpersonal dimensions like support) for diabetics [85], and a questionnaire (about autonomy supportive communication) in breast cancer care [87]. Also, associations were examined twice. They regarded perceived support, anxiety, and device acceptance among implantable cardioverter defibrillator recipients [86], and perceived autonomy‐supportive communication and decision quality in breast cancer care [88]. Physicians' communication behaviours (such as supportiveness) concerning ethnicity was studied by post‐consultation questionnaires [88]. Experiences were studied only once qualitatively. This study dealt with supportive communication in ulcerative colitis visits and disease's social and emotional impacts [89]. *Together, expectations and experiences* were studied as (support) gaps in the case of infertility by data from online surveys [56]. *Actual supportive behaviours* during visits were examined only twice, both by audio records. These studies explored patient participation with physician's communication styles (such as partnership‐building and supportive talk) [79], and how to facilitate patients' involvement by them [80] (Tables 4, 5).

### 3.2 Existing Knowledge About Supportive Patient–Provider Communication in Question

Studies about *patients' expectations* concerned mainly cancer care. In a study about end‐of‐life care also patients with AIDS and COPD were included [82]. End‐of‐life patients' essential expectations concerned emotional support and communication with accessibility to and continuity of care. Expectations were largely analogous while some disease‐specific differences existed. Patients with COPD stressed the importance of information about diagnosis, disease process, treatment, prognosis, advance care planning, and dying. Patients with cancer longed for support to maintain hope. Patients with AIDS stressed the significance of having access to pain relief by a physician who do not hesitate to prescribe efficient medication. All expected emotional, informational, and tangible support, albeit COPD patients highlighted informational, cancer & AIDS patients emotional, and patients with AIDS tangible support [82] (Tables 4, 5).

In a cancer study about learning and support preferences patients desired clear, accurate, and interactive information. They preferred discussions with physicians, although also printed information from providers, discussions with nurses, print media, and conversations with other patients (in this order) were welcomed. However, learning preferences varied significantly by age, gender, and education [83]. Breast cancer susceptibility (BRCA‐positive) women expected supportive communication (emotional support), information, partnership in decision making, and guidance about social support networks for uncertainty management. Thus, it seems reasonable to say that they sought emotional, informational, tangible, and network support [84] (Tables 4, 5).

Studies about *patients' experiences* concerned diabetes, heart disease, breast cancer, ulcerative colitis, and primary care. Diabetics experienced interpersonal factors, such as empathy and communication, as more important than organisational factors. Those of them who reported poor mental health were significantly less satisfied with the interpersonal quality of their care than those experiencing good mental health. As for patients with poor general health, they were significantly less satisfied with the organisational aspects than patients with good health [85]. In regular follow‐up visits, constructive support increased device acceptance. Providing understandable information and allowing sufficient time to process emotions were essential for that. By contrast, non‐constructive support—such as indifference, criticism, and information that may lead to insecurity and fear—may cause distress and reduce device acceptance [86] (Tables 4, 5).

Patients with breast cancer differentiated their perceptions about autonomy support according to providers' specialty. This indicates, that specialist‐specific ratings could be utilised for more detailed information [87]. Further, in the case of breast‐cancer, autonomy‐supportive communication was associated with higher subjective decision quality. This association between (received) physician communication style and (perceived) decision quality was moderated by preferred patient communication style (directive or non‐directive like autonomy‐supportive) [88]. Patients with ulcerative colitis felt commonly emotional impacts such as depression and anxiety, decreased self‐confidence, lack of motivation, anger, frustration, and embarrassment. These emotional burdens may be notable even with low symptoms. Most patients reported that their physician asked about their symptoms and listened to them. However, many of them perceived that emotional support was lacking [89] (Tables 4, 5).

One study about physicians' communication behaviour located in rushed primary care in Bangladesh. Patients experienced more supportive communication when their ethnicity/race was concordant with providers revealing disparities in care. Supportive communication behaviours included, for example, cheerful greetings; encouragement to express health problems and ask questions; careful listening; responding to questions and concerns; explaining about procedures, medication, probable side effects; discussing treatment options; involving patients in decisions; and spending adequate time. The factors associated with patients' experience of better communication diverged between ethnic minority and majority groups. However, having a pre‐organised plan about the content of visit benefitted all patients [81] (Tables 4, 5).

Studies about *patients' expectations and experiences together* [56] concerned the support that infertile women desire and receive. Significant discrepancies existed not only between the amount but also the types of supportive communication. These women received emotional, network, tangible, and even informational support less than desired. The magnitude of the gap between desired and received support reflected evaluations of support quality [56] (Tables 4, 5).

*Actual behaviours* about supportive communication were studied only twice. This may originate from burdensome and laborious methods, which are common during data generation and analyse. Both studies were quantitative, from the US, and partly from the same researchers. They concerned patients' participation and its' facilitation in diverse settings (by health conditions, medical specialty, and allocated time). *Supportive talk* (empathy, encouragement, praise, and reassurance) occurred in less than 40% of interactions. Female physicians offered it significantly more than males. Supportive talk predicted patients' expressions of concern and assertiveness but not question‐asking. As for *partnering behaviour* (verbal acts that either encouraged patients' involvement or affirmed or accommodated their active participation), it occurred generally at least once in visit. Patients were significantly more active when physicians facilitated communication by partnership building and supportive talk. However, active participation was mostly patient initiated, not a cause of facilitation. More active patients, who were also more educated and more likely whites, received more facilitative communication. Clinical setting (such as health condition, medical specialty, the scheduled length of the visit) and the physician's communicative style were the strongest predictors for patient participation [79]. One study was organised in two diverse post‐diagnosis settings, where treatment options were discussed. In both cases, the proportion of partnership‐building and supportive talk was the same despite the differences in visit length, specialty, and outpatient or inpatient status. Additionally, patients engaged more when physicians' talk was proportionally more facilitative [80] (Tables 4, 5).

## 4 Discussion

The aim of this review ([67, 68]) was to examine *supportive patient‐provider communication* in outpatient visits. We did that by inspecting behaviours, and patients' expectations and experiences *from the communication perspective of social support* [45]. We aspired to enlighten the extent, range, and nature of such studies, and determine what is known about such supportive communication. Now, we continue with discussion about the studies, findings, and research gaps.

Existing knowledge about *supportive patient‐provider communication* in outpatient visits proved to be surprisingly scarce considering research activity around healthcare and patient‐provider interaction at large. Regardless of the wide scope in time, journals, locations, specialties, diseases, patients, and providers, only handful of studies *from the communication perspective of social support* emerged. What could possibly explain this while knowing the evidence about positive effects of supportive communication concerning psychological and physical health? [45].

Maybe medical history about ‘placebo’ could serve some answers. Namely, long‐ago physicians noticed that sometimes patients' health improved without any known active substance in medical treatment. This unknown element (‘placebo’) was judged as ‘non‐treatment’, even as sham, while its positive effects were perceived as inappropriate (‘placebo effects’). Since, medical treatments have been strictly safeguarded from non‐medical influences. Patient‐provider communication, inter alia, include in ‘placebo’ labelled and thus its' effects irrelevant. Only lately this perspective has been questioned with demands to acknowledge also non‐medical evidence, and reframe it in a more respectful manner [97, 98, 99]. In a way, it is understandable that communication has been seen as irrelevant from medical perspective, as it is not, indeed, medical treatment. However, it would be finally time to legitim existing evidence about communication as valuable, beneficial, and highly applicable in healthcare along with various treatments and therapies.

Further, the fact that (supportive) outpatient‐provider communication is interdisciplinary by nature, may complicate every step also during research process restraining their amount. For example, funders focused on healthcare may see that it is not their duty to fund communication studies. Funders focused on communication, in turn, may consider that healthcare specified communication belong to healthcare. Then, when aiming to report studies, interdisciplinarity restrain again substantially as there are only few potential academic journals with adequate scope and reviewers.

Paucity of studies make existing knowledge inevitably fragmented. This will be partly relieved along growing study mass. However, multiplicity in central concepts and conceptualisations originating from interdisciplinarity, is challenging and confusing. Variety of conceptualisations concerning expectations [58, 59] and experiences [100] within healthcare has already noted (see also [64]). Diversity of central concepts is also well‐known (e.g., patient‐centred communication [101, 102] and patient‐centeredness [103]). Thus, efforts towards greater congruence and clarity are extremely important.

Existing knowledge proved to be also unilateral. Currently, it is mainly based on quantitative surveys and studies about whites/Caucasians, specialised physicians, and patients with cancer in North America (US). This is problematic, as according earlier knowledge, we know that supportive communication is affected by many factors including provider, receiver, and context [54]. Therefore, we do not know, if and how the findings would differ for example in the case of primary care physicians or other providers, patients, and cultures. Besides, obviously, we need both quantitative and qualitative studies for broad and profound understanding.

Though, the prominence of US reflects research activity in the fields of communication (and health related) sciences. The prominence of physicians suggests that they are valued as more important than other providers. However, supportive communication should be studied more broadly, as for example in the case long‐term illnesses, self‐management support is largely provided by nurses and physiotherapists [78]. Focus on specialised physicians and patients with cancer may derive from cancer's prevalence, disease's severity, and required resources regarding healthcare and society [104]. Delivering devasting news and explaining complex treatments during brief visits is challenging [105]. However, situations with devasting news, and tight time frames are common also to other physicians [19, 106].

Context, including time and provider resources, is significant regarding outpatient‐provider communication. Here, when reported, visit duration ranged from less than 5 min up to 30 min. Earlier, the mean duration of ambulatory cancer visits has been reported to be 22.9 min (in America) in years 2006–2007 [104]. According to systematic review of 67 countries with data from year 1946 to 2016 primary care physicians' average visit duration ranged from 48 s (in Bangladesh) to 22.5 min (in Sweden). Time for patient‐provider communication was only 5 min or less in 18 countries covering ~50% of the world's population [19]. Recently the average visit duration in primary care was 9.10 min in Bangladesh [107] corresponding quite well to reported duration in this review [81]. Here, discrepancy between patients in need of care and economic resources for providing sufficient care was mentioned by long lines of waiting patients, busyness of physicians [81], and difficulties in providing preferred learning methods because of time pressures [83]. While patients are justified to receive supportive communication, providers are justified to sufficient resources for that. Thus, supportive communication is not just a matter of patients and providers, it is a matter of organisations, systems, and societies [12].

Expectations are known to have considerable influence both on physical and mental health, and medical treatment outcomes [62, 63]. Some patients may, for example expect that as physicians are busy, support is not available, while others may expect that they are entitled to valuable support. In both cases, expectations may effect on their experiences [108]. Here, *patients' expectations* were studied only as behaviours *valued* by patients [58, 59, 61]. However, as patients' future‐directed beliefs—expectations as probabilities—are predictive by nature [62], understanding about both expectations for support and health (see [109]) is relevant to enhance outcomes [56]. As for outpatients' desire for support through access to and continuity of care [82], healthcare providers have only limited power to respond them. However, access to healthcare, consultation time, and continuity of care enable or constrain the support needed in chronic conditions [37]. Therefore, organisations/systems should be perceived as integral (f)actors towards supportive outpatient‐provider communication.

Studies about *patients' experiences* concerned typically ongoing care instead of single visits, although more nuanced qualitative knowledge about *communication* could be achieved by them. Focusing on certain type of visits could be another option for more specific knowledge (see [87]). Though, the findings provide us valuable knowledge. Making a pre‐organised plan is a usable tool for patients [81] whereas providers can utilise autonomy‐supportive communication to improve decision quality [88]. Further, understanding that chronic diseases may cause noteworthy negative emotional impacts with support needs, although the physical symptoms would be mild [89] is important for clinicians. The fact, that while clinician's communication can be more or less supportive, provided support can be also nonconstructive (e.g., indifference, criticism, and information that leads to insecurity or fear) [86] highlights the need for understanding patients' experiences.

Based on the findings about *patients' expectations and experiences together*, we know now that the desired amount and type of supportive communication, may differ significantly from experienced ones. The gap between patients' desires and experiences seems to correspond with evaluations of support quality. Thus, studies about expectations (as values see [59, 61]) and experiences together are necessary also in future.

Lastly, the findings about *actual supportive communication* remind us about factors' variety. While female physicians tend to offer more supportive talk than males, also patients' features such as activity and ethnicity/race affect the amount providers use supportive talk with them [79]. Further, patient involvement can be facilitated by partnering and supportive communication [79, 80]. As patients' participation also depends on contextual factors [79, 80], diverse data are needed.

As stated, the knowledge gaps regarding reviewed subject are manifold. While considerably knowledge about social support studied from sociological and psychological perspectives exists, likewise supportive and patient‐provider communication more widely, they were out of scope in this review. However, they are valuable resources when striving to increase more specific knowledge from communication perspective. While recognising the shortages in knowledge, we should also recognise the shortages, which impede first research, then healthcare providers to exploit existing knowledge. Specifically, shortage of resources seems to be a common problem. Further, clinicians need to learn skills to provide supportive messages, adapt them based on needed quantity, quality, and type of support, and reflect both their own and patients' communication in practice. Moreover, when aiming to supportive outpatient‐provider communication, organisations are responsible to provide support both for providers and patients. Therefore, along with studies about supportive outpatient‐provider communication, we need to strive for recourses, education, management, and organisations to make supportive communication achievable for healthcare providers and their patients (see [38]).

A primary limitation of scoping reviews is the absence of distinct quality appraisal. Here, the fact that all studies were passed peer‐review process before publishing was utilised as quality requirement. Considering the purpose of this review, a scoping review can be seen adequate by enabling to deal with a wide range of studies, map their nature, extent, and variation, summarise findings, identify gaps, and draw conclusions (see [67, 68]). Our search strategy included four communication and health research databases with English journal articles focusing specifically on the *communication perspective*—not on social support as a whole or other perspectives [45], which were out of scope. Some relevant studies from other databases, fields, and languages may have missed because of these decisions. Building an appropriate search strategy was quite challenging because of differences between databases, disciplines, studies, and concepts. Thus, we consulted university informatician. Further, preliminary searches with various search strategies were profound with iterative evaluations and modifications. Moreover, we updated search during the reporting process to enhance the timeliness. After diligent preparations, the study selection proceeded quite smoothly with the guidance of research questions and selection criteria. The first author screened all the data in a rigorous and transparent way through discussions about observations with other authors, including explicit documentation to foster reliability [67]. Nevertheless, interpretation of the included studies was subject to reviewer bias and in case of more resources multiple researchers would have screened all the data. Finally, we must notice the limited quantity of studies, as for their one‐sidedness concerning culture, research subjects, and methods.

## 5 Conclusion

This review focused on the communication perspective of social support [45], and contributed to existing knowledge by summarising the studies and findings, and by identifying research gaps. According to existing data, actual evidence about supporting outpatient‐provider communication is still surprisingly thin and unilateral both as actual supportive behaviours, and patients' expectations and experiences. Thus, the research gaps proved to be manifold.

As the current knowledge is mainly based on few quantitative surveys and studies about whites/Caucasians, specialised physicians, and patients with cancer in North America (US), we must be cautious when drawing conclusions from study findings. In future studies, we need more diversity in patients, providers, clinical settings, cultures, and methods. While both quantitative and qualitative methods are important, the paradigm of person‐centeredness obligate to examine patients' perspective especially by qualitative approach. Particularly, video materials from actual behaviours, as well expectations (as values) together with experiences are lacking. As future‐directed beliefs are predictive by nature, studies about chronically ill patients' expectations as probabilities would be worthwhile when aiming to enhance their self‐management support and health outcomes.

Supportive outpatient‐provider communication is not just a matter of patients and providers, it is a matter of organisations, systems, and societies. Therefore, they should be perceived as integral (f)actors of supportive outpatient‐provider communication in research, education, and practice. Further, we need interdisciplinary research collaboration to enhance, deepen and broaden our understanding about this subject and to facilitate conceptual clarity. The knowledge about supportive, person‐centred, and patient‐provider communication serves us a valuable ground until we get more specific knowledge about supportive outpatient‐provider communication in visits.

## Author Contributions

**Sari Knaapi‐Junnila:** funding acquisition, conceptualisation, methodology, investigation, writing – original draft, visualisation. **Tuula‐Riitta Välikoski:** funding acquisition, aupervision, writing – review and editing. **Pekka Isotalus:** funding acquisition, supervision, writing – review and editing.

## Ethics Statement

The authors have nothing to report.

## Consent

The authors have nothing to report.

## Conflicts of Interest

The authors declare no conflicts of interest.

## Data Availability

Data sharing not applicable to this article as no datasets were generated or analysed during the current study.
